# Supplementary material for: Use of a modular ontology and a semantic annotation tool to describe the care pathway of patients with amyotrophic lateral sclerosis in a coordination network
Source: PLoS One. 2021 Jan 6;16(1):e0244604. doi: 10.1371/journal.pone.0244604 (PMC7787442; doi:10.1371/journal.pone.0244604)
Supplement: S1 Table — List of some of the defined concepts and their formal definitions, present in each module of the OntoPaRON modular ontology. We recall that the HermiT reasoner infers the membership of all concepts sharing a relationship to a fully defined concept defined with this same relationship. (PDF) [file pone.0244604.s001.pdf]

S1 Table: List of some of the defined concepts in OntoPaRon Ontology.

Module	Defined concept	Formal definition
**Core module**	‘accessibility problem’	‘abstract object’ and *has a thematic* some ‘thematic notion of accessibility issues’
	‘area of need and request for medical care’	‘abstract object’ and *has a thematic* some ‘thematic concept of medical needs and demands’
	‘area of need and request for social help’	‘abstract object’ and *has a thematic* some ‘thematic concept of needs and demands for social care’
	‘area social problem’	‘abstract object’ and *has a thematic* some ‘thematic notion of social problem’
	‘field of exhaustion’	‘abstract object’ and has *a thematic* some ‘thematic concept of exhaustion’
	‘field of human help’	‘abstract object’ and *has a thematic* some ‘thematic concept of human aid ’
	‘field of technical aids’	‘abstract object’ and *has a thematic* some ‘thematic concept of technical devices’
**Coordination module**	‘coordination action’	‘action’ and *has a thematic* some ‘thematic concept of pathway coordination’
	‘coordination communication action’	‘action of communicating’ and *has a thematic* some ‘thematic concept of pathway coordination’
	‘outbound communication action’	‘coordination communication action’ and *has a thematic* some ‘thematic outgoing communication’; and *has a thematic* some ‘thematic concept of pathway coordination’
	‘request received in coordination’	‘action of requesting’ and *has a thematic* some ‘thematic concept of pathway coordination’
	‘matching resources to needs’	‘guiding activity’ and *has a thematic* some ‘thematic concept of pathway coordination’
	‘advocacy prevention coordination’	‘advocate’ and *has a thematic* some ‘thematic concept of pathway coordination’
**Medical module**	‘medical action’	‘action’ and *has a thematic* some ‘medical thematic concept’
	‘medical assessment’	‘evaluating’ and *has a thematic* some ‘thematic concept medical assessment’

	‘paramedical evaluation’<br><br>‘cognitive state’<br><br>‘cutaneous state’<br><br>‘medical condition’<br><br>‘motor state’<br><br>‘neurological impairment’<br><br>‘nutritional condition’<br><br>‘painful condition’<br><br>‘psychological state’<br><br>‘respiratory condition’<br><br>‘treatment-related condition’	‘evaluating’ and *has a thematic* some ‘thematic concept paramedical evaluation’<br><br>‘status’ and *has a thematic* some ‘cognitive-behavioural thematic concept’<br><br>‘status’ and *has a thematic* some ‘skin thematic concept’<br><br>‘status’ and *has a thematic* some ‘medical thematic concept’<br><br>‘status’ and *has a thematic* some ‘thematic concept, driving and functional’<br><br>‘status’ and *has a thematic* some ‘neurological thematic concept’<br><br>‘status’ and *has a thematic* some ‘thematic concept of nutrition’<br><br>‘status’ and *has a thematic* some ‘thematic notion of pain’<br><br>‘status’ and *has a thematic* some ‘psychic thematic concept’<br><br>‘status’ and *has a thematic* some ‘breath thematic concept’<br><br>‘status’ and *has a thematic* some ‘thematic concept treatment’
**Socio-environmental module**	‘social process’<br><br>‘social action’<br><br>‘social state’	‘process’ and *has a thematic* some ‘social thematic concept’<br><br>‘action’ and *has a thematic* some ‘social thematic concept’<br><br>‘status’ and *has a thematic* some ‘social thematic concept’
